# The let-7b-5p, miR-326, and miR-125a-3p are associated with left ventricular systolic dysfunction in post-myocardial infarction

**DOI:** 10.3389/fcvm.2023.1151855

**Published:** 2023-05-12

**Authors:** Raquel Costa Silva Dantas-Komatsu, Marina Sampaio Cruz, Paula Paccielli Freire, Rosiane Viana Zuza Diniz, Raul Hernandes Bortolin, Otávio Cabral-Marques, Kamilla Batista da Silva Souza, Mario Hiroyuki Hirata, Rosario Dominguez Crespo Hirata, Bruna Zavarize Reis, Igor Jurisica, Vivian Nogueira Silbiger, Andre Ducati Luchessi

**Affiliations:** ^1^ Postgraduate Program in Health Sciences, Federal University of Rio Grande do Norte, Natal, Brazil; ^2^ Division of Cardiology, Department of Medicine, UC San Diego, San Diego, CA, United States; ^3^ Department of Immunology, Institute of Biomedical Sciences, University of São Paulo, São Paulo, Brazil; ^4^ Department of Clinical Medicine, Center for Health Sciences, Federal University of Rio Grande do Norte, Natal, Brazil; ^5^ Department of Clinical and Toxicological Analyses, School of Pharmaceutical Sciences, University of Sao Paulo, São Paulo, Brazil; ^6^ Department of Cardiology, Boston Children’s Hospital/Harvard Medical School, Boston, MA, United States; ^7^ Division of Molecular Medicine, Department of Medicine, University of São Paulo School of Medicine, São Paulo, Brazil; ^8^ Laboratory of Medical Investigation, University of São Paulo School of Medicine, São Paulo, Brazil; ^9^ Interunit Postgraduate Program on Bioinformatics, Institute of Mathematics and Statistics, University of Sao Paulo, Sao Paulo, Brazil; ^10^ Department of Nutrition, Center for Health Sciences, Federal University of Rio Grande do Norte, Natal, Brazil; ^11^ Division of Orthopedic Surgery, Schroeder Arthritis Institute and Data Science Discovery Centre for Chronic Diseases, Krembil Research Institute, University Health Network, Toronto, ON, Canada; ^12^ Departments of Medical Biophysics and Computer Science, and Faculty of Dentistry, University of Toronto, Toronto, ON, Canada; ^13^ Slovak Academy of Sciences, Institute of Neuroimmunology, Bratislava, Slovakia; ^14^ Department of Clinical and Toxicology Analysis, Federal University of Rio Grande do Norte, Natal, Brazil; ^15^ Translational Medicine, The Hospital for Sick Children (SickKids), Toronto, ON, Canada

**Keywords:** miRNA, PBMC, myocardial infarction, left ventricular systolic dysfunction, heart failure, enrichment analysis

## Abstract

**Background:**

Acute ST-elevation myocardial infarction (STEMI) can lead to adverse cardiac remodeling, resulting in left ventricular systolic dysfunction (LVSd) and heart failure. Epigenetic regulators, such as microRNAs, may be involved in the physiopathology of LVSd.

**Objective:**

This study explored microRNAs in peripheral blood mononuclear cells (PBMC) of post-myocardial infarction patients with LVSd.

**Methods:**

Post-STEMI patients were grouped as having (LVSd, *n* = 9) or not LVSd (non-LVSd, *n* = 16). The expression of 61 microRNAs was analyzed in PBMC by RT-qPCR and the differentially expressed microRNAs were identified. Principal Component Analysis stratified the microRNAs based on the development of dysfunction. Predictive variables of LVSd were investigated through logistic regression analysis. A system biology approach was used to explore the regulatory molecular network of the disease and an enrichment analysis was performed.

**Results:**

The let-7b-5p (AUC: 0.807; 95% CI: 0.63–0.98; *p* = 0.013), miR-125a-3p (AUC: 0.800; 95% CI: 0.61–0.99; *p* = 0.036) and miR-326 (AUC: 0.783; 95% CI: 0.54–1.00; *p* = 0.028) were upregulated in LVSd (*p* < 0.05) and discriminated LVSd from non-LVSd. Multivariate logistic regression analysis showed let-7b-5p (OR: 16.00; 95% CI: 1.54–166.05; *p* = 0.020) and miR-326 (OR: 28.00; 95% CI: 2.42–323.70; *p* = 0.008) as predictors of LVSd. The enrichment analysis revealed association of the targets of these three microRNAs with immunological response, cell-cell adhesion, and cardiac changes.

**Conclusion:**

LVSd alters the expression of let-7b-5p, miR-326, and miR-125a-3p in PBMC from post-STEMI, indicating their potential involvement in the cardiac dysfunction physiopathology and highlighting these miRNAs as possible LVSd biomarkers.

## GRAPHICAL ABSTRACT

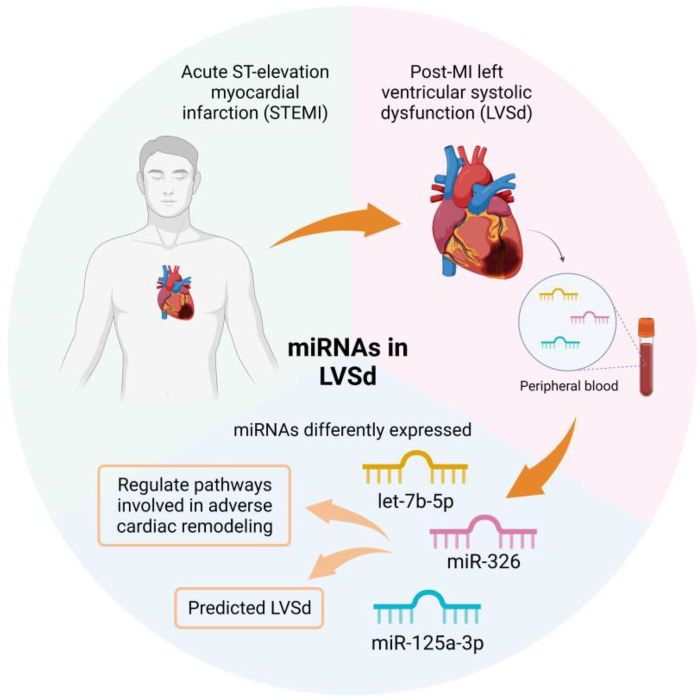

## Introduction

Acute myocardial infarction (AMI) is a severe cardiovascular disease leading to progressive cardiac remodeling (1). Cardiac remodeling is a compensatory, adaptive, and functional process in response to a given aggression. When sustained, it can progress to adverse remodeling, with structural changes in cardiomyocytes, endothelium, smooth muscle cells, interstitial cells, and extracellular matrix (2). Potential factors involved in cardiac remodeling are energetic metabolism disturbance, oxidative stress, inflammation, neurohormonal activation, contractility reduction of cardiomyocytes, calcium transport imbalance, cell death, and fibrosis (3).

The adverse remodeling majority occurs during the first 3–6 months following ST-segment elevation myocardial infarction (STEMI) (4) and reaches approximately 40% of patients with STEMI (5). This pathological remodeling plays an essential role in the development of left ventricular systolic dysfunction (LVSd) and subsequent heart failure (HF) (2), which is a source of concern given that 70% of patients who develop HF after AMI die within 7.6 years (6). Therefore, the prediction of LVSd in post-myocardial infarction (post-MI) patients is essential to provide treatment that attenuates or even reverses the process and improves poor outcomes (7).

The detection of morphological changes in the clinical diagnosis of ventricular dysfunction is still based on cardiac imaging tests such as echocardiography and nuclear magnetic resonance (3). At the same time, molecular biomarkers are limited to proteins that indicate cardiac hemodynamic stress (e.g., N-terminal pro-B-type natriuretic peptide) or cardiomyocyte damage (e.g., cardiac troponin) (8). Despite study advances, there is still a need to understand the molecular mechanisms of left ventricular remodeling and to find early biomarkers for accurate LVSd detection and therapeutic targets to prevent the development of HF.

Among the molecular network involved in HF, microRNAs (miRNAs) are implicated in various pathological processes in the post-MI heart, suggesting that their up- or downregulation modulates metabolic pathways related to cardiac remodeling and HF (9). miRNAs are small non-coding RNAs that regulate gene expression by post-transcriptionally binding to the 3′-untranslated region of target mRNAs and inducing gene silencing by translational inhibition or mRNA degradation (10, 11). Several studies support the potential of miRNAs as physiological biomarkers, therapeutic mediators, and even as a predictor of therapeutic efficacy or poor prognosis of various immune-mediated diseases (12), such as cancer (13), “Alzheimer's disease” (14), and HF (15).

Of note, the immune system plays an essential role in cardiac disease (16) and miRNAs of peripheral blood mononuclear cells (PBMC) have been shown to be dysregulated in cardiovascular diseases (17). Thus, these molecules may be used as biomarkers to identify patients with atherosclerotic coronary artery disease at risk of acute coronary syndromes (18) or even to differentiate HF patients from healthy subjects (19). In this study, the expression of miRNAs was assessed in PBMC of post-MI patients with LVSd, and a system biology approach was used to explore the regulatory molecular network of the disease, focusing on pathways involved with cardiac remodeling, cell-cell communications, and immunological changes.

## Materials and methods

### Study subjects

Patients who experienced STEMI at least two months before enrollment and were treated in Onofre Lopes University Hospital of the UFRN, Brazil, between July 2018 and December 2019 were eligible for this cross-sectional study. The patients were recruited during their routine appointment at the Cardiology Outpatient Unit. The exclusion criteria were other causes of LVSd or HF, such as congenital cardiomyopathies, dilated cardiomyopathy, atrial fibrillation, Chagas disease, and non-treated hypertension. Patients who were referred for cardiac transplants were also excluded. Demographic, clinical, and echocardiographic characteristics were obtained from electronic medical records and personal interviews. The sex of participants was defined based on self-report and was used as a classification of male or female based on biological distinction. The clinical outcome of the post-MI patients was determined by left ventricular ejection fraction (LVEF) measured at ± three months from the date of venous blood collection. Patients were grouped as having LVSd (LVEF ≤ 40%) or not (non-LVSd, LVEF > 40%) (20).

### Study approval

The Ethics Committees of the Federal University of Rio Grande do Norte (UFRN), Brazil (CAAE #65856317.1.0000.5292) approved the study protocol. The study was conducted according to good clinical practices and the Helsinki guidelines (as revised in 2013). All subjects signed an approved written informed consent before enrollment. All experiments were performed following relevant guidelines and regulations.

### Biochemical and hematological measurements

Hemoglobin concentration, hematocrit, leukocytes, and platelet numbers were measured in whole blood samples using an automated XT-2000i (Sysmex Corporation, Kobe, Japan). Fasting serum glucose, total cholesterol, high-density lipoprotein cholesterol, low-density lipoprotein cholesterol, triglycerides, sodium, potassium, urea and creatinine concentrations, alanine aminotransferase, and aspartate aminotransferase enzymatic activity were measured using Wiener kits and a CMD-800 automatic biochemistry analyzer (Wiener Laboratories, Rosario, Argentina).

### miRNA expression

PBMC were isolated from 4 ml of whole blood in EDTA using ammonium chloride as lysis solution and were stored in Trizol (Invitrogen, Waltham, MA, USA) at −80°C until analysis. Total RNA was extracted using Trizol according to the “manufacturer's protocol”. RNA concentration and purity were measured using a Nanodrop spectrophotometer (NanoDrop ND-1000, Montchanin, DE, USA). RNA integrity was determined by agarose gel electrophoresis (2% agarose/MOPS). The gel was stained with GelRed (Uniscience, São Paulo, SP, Brazil), revealing the presence of two sharp bands at approximately 5 and 1.8 Kb, corresponding to 28S and 18S ribosomal RNA, respectively.

The reverse transcription of miRNA samples was performed using TaqMan™ Advanced miRNA cDNA Synthesis Kit (Applied Biosystems, Waltham, MA, USA). For the analysis, 61 miRNAs previously described in the literature were selected for their relationship with cardiovascular diseases (see Supplementary Table S1). The miRNAs' expression was measured by Real-Time quantitative PCR (RT-qPCR) using a custom TaqMan™ Advanced miRNA Human A and B 96-well Plates, TaqMan™ Fast Advanced Master Mix Kit (Applied Biosystems, Waltham, MA, USA) and were performed in QuantStudio™ 5 Real-Time PCR System, 96-well. No miRNA showed cycle threshold (Ct) values higher than 35 in the analysis. Relative miRNA levels were measured using the comparative Ct method (2^−ΔΔCt^ for calculate the fold-change value; and 2^−ΔCt^ for other statistical analysis) (21) normalized by miR-16-5p as a reference, a miRNA abundantly expressed in all tissues and commonly used as a control (22, 23). The miRNAs differentially expressed in the LVSd, as compared with the non-LVSd, were selected considering a fold-change of |1.5| and *p*-value < 0.05 (see Supplementary Table S1).

### Principal component analysis

Principal Component Analysis (PCA) was used to stratify the miRNAs based on the post-MI patients who developed LVSd or not. Before the analysis, the miRNAs expression data were log2-transformed. PCA was performed using the R package factoextra and the prcomp function, in which data was centered and scaled. The number of principal components was chosen according to Kaiser Criterion. References for all statistical tools used in this study are listed in Supplementary Table Si.

### Prediction of miRNAs targets

The target genes of differentially expressed miRNAs were identified using the microRNA Data Integration Portal (mirDIP ver. 5.2; https://ophid.utoronto.ca/mirDIP). All annotated interaction data are presented in Supplementary Material. In addition, the miRTarBase 9.0 (https://mirtarbase.cuhk.edu.cn/) and DIANA TOOLS-Tarbase ver.8 (https://dianalab.e-ce.uth.gr/) were used to select the experimentally validated miRNA-mRNA interactions. Only interactions validated by robust evidence methods (reporter assay, western blot, and qPCR) were selected. References for all bioinformatics tools used in this study are listed in Supplementary Table Si.

### Enrichment analysis and data visualization

The experimentally validated miRNA target genes were used for enrichment analysis. Biological processes (GO) were analyzed using EnrichR (http://amp.pharm.mssm.edu/Enrichr/), and the enriched terms were filtered according to adjusted *p*-value < 0.05 and Z-score (correction to the test) in a combined score provided by EnrichR database. The biological process terms presented in the bubble heatmap were selected based on the criterion of overrepresentation (log2 combined score >2) in at least two miRNAs. Concomitantly, we searched for the enriched pathways using the Kyoto Encyclopedia of Genes and Genomes (KEGG) using Database for Annotation, Visualization and Integrated Discovery (DAVID ver.2021; https://david.ncifcrf.gov/). We selected the pathways with a *p*-value threshold < 0.05 after correction for False Discovery Rate (FDR). Only experimentally validated miRNA-mRNA interactions were used for this analysis.

We plotted the biological processes associated with the three differentially expressed miRNAs (let-7b-5p, miR-326, miR-125a-3p) in a bubble-based heat map with hierarchical clustering using the web tool Morpheus (https://software.broadinstitute.org/morpheus/) with Euclidian distance metric. We created a mirrored bar plot using the GraphPad Prism ver.9.2 (GraphPad Software, Inc., San Diego, CA, USA) to represent the enriched pathways from the KEGG.

### Molecular network of miRNA-mRNA interactions

The miRNA-gene network was prepared using mirDIP ver. 5.2 (https://ophid.utoronto.ca/mirDIP), considering all sources and top 1% gene targets. Only shared targets are highlighted with names, but all gene targets are annotated with Gene Ontology biological process, as per color legend (Figure 3A). Also, shared mRNAs target genes among three miRNAs were displayed using the Circos software ver. 0.63–10 (http://circos.ca/) (see Supplementary Figure S1).

Taking advantage of curated tissue annotation for both miRNA and gene targets, Figure 3B highlights tissues associated with the three miRNAs. To further characterize biological relationships among the three miRNAs, we have generated a physical protein interaction network among miRNA targets using Integrated Interactions Database (IID ver. 2021-05; http://ophid.utoronto.ca/iid). All direct human interactions among the target genes are shown, highlighting those relevant to heart and cardiovascular disease (edge color as per legend). We differentiate whether only one of the interactors has the annotation (partially transparent edge with color as per legend) or both interacting partner share the annotation (solid edge with color as per legend), and whether the annotation is valid for both heart and cardiovascular system disease (strong solid edge with color as per legend). In addition, node color corresponds to Gene Ontology biological process. All annotated interaction data are presented in Supplementary Material.

All networks were obtained using NAViGaTOR ver 3.0.16. Network images were exported in SVG format into Adobe Illustrator ver. 27 to prepare final figures with legends.

### Statistical analysis

The distribution of the continuous variables was analyzed using the Shapiro-Wilk test. Variables with normal distribution are shown as mean and standard deviation and were compared by *t*-test for independent variables. Those with skewed distributions are shown as median and interquartile range and compared using Mann-Whitney *U*-test. Spearman's correlation tests assessed the correlation analysis between differentially expressed miRNAs. Categorical variables were compared using Fisher's exact test and are shown as relative and absolute frequency.

Assessing the discriminatory power of the selected miRNA as an LVSd predictor, a receiver operating curve (ROC) was constructed, and the area under the curve (AUC) was calculated with a 95% confidence interval (CI). The cut-off point for miRNA was identified based on an excellent combination of sensitivity and specificity (AUC ≥ 0.7).

Univariate logistic regression analysis was performed to evaluate the variables that predict post-MI LVSd. Multivariate logistic regression analysis was performed using a forward stepwise method only with statistically significant miRNAs in the univariate analysis and adjusted by sex, body mass index, smoking, alcohol intake, diabetes, and time from AMI to enrollment as independent variables.

Statistical analysis was performed using SPSS Statistics V23.0 software (IBM, IL, USA), GraphPad PRISM, version 5.0 (GraphPad Software, Inc., San Diego, CA, USA), or R program (v4.1) using packages ggplot2 (v3.4.0) and the *p*-value < 0.05 was considered statistically significant.

## Results

### Patient characteristics

The description of clinical characteristics of post-MI patients is shown in Table 1. Most of the patients enrolled in the study were men, and 36% developed LVSd. They presented LVEF < 40%. The groups did not differ in terms of age, BMI, chronic diseases, and habits such as physical activity, alcohol intake, and smoking, as well as concerning biochemical data (Table 2). This similarity between the participants was expected because both groups are composed of people who have already suffered STEMI and were undergoing medical follow-up.

**Table 1:** Clinical characteristics of post-MI patients.

Variables	Total (25)	non-LVSd (16)	LVSd (9)	*p*-value
Age, years	57 ± 9	55 ± 9	61 ± 9	0.125
Sex, male	80.0 (20)	81.3 (13)	77.8 (7)	1.000
Smoking	60.0 (15)	62.5 (10)	55.6 (5)	1.000
Alcohol intake	68.0 (17)	75.0 (12)	55.6 (5)	0.394
Physical activity	76.0 (19)	75.0 (12)	77.8 (7)	1.000
BMI, kg/m²	28.6 ± 4.9	28.8 ± 4.9	28.4 ± 5.1	0.853
Obesity	36.0 (9)	31.3 (5)	44.4 (4)	0.671
Diabetes	40.0 (10)	25.0 (4)	66.7 (6)	0.087
Hypertension	80.0 (20)	68.8 (11)	9 (100)	0.123
Dyslipidemia	100 (25)	100 (16)	100 (9)	—
LVEF, %	45.6 ± 14.1	54.1 ± 9.7	30.4 ± 3.7	**0**.**010**
Systolic blood pressure, mmHg	120 (110–125)	120 (110–120)	120 (110–130)	0.907
Diastolic blood pressure, mmHg	80 (70–85)	80 (70–85)	80 (70–90)	0.953
Heart rate, beats/min	69 ± 13	69 ± 14	70 ± 12	0.982
Family history of AMI	72.0 (18)	75.0 (12)	66.7 (6)	0.673
Time from AMI to enrollment, days	291 (215–999)	251 (172–399)	999 (256–1,771)	**0**.**027**
**Medications**				
ACEI/ARA	100 (25)	100 (16)	100 (9)	—
β-blockers	100 (25)	100 (16)	100 (9)	—
Diuretics	52.0 (13)	31.3 (5)	88.9 (8)	**0**.**011**
Antidiabetics	32.0 (8)	18.8 (3)	55.6 (5)	0.087
Vasodilators	32.0 (8)	12.5 (2)	66.7 (6)	**0**.**010**
Antiplatelet agents	100 (25)	100 (16)	100 (9)	—
Statins	100 (25)	100 (16)	100 (9)	—

Bold indicates a significant *p*-value (< 0.05).

The number of subjects is shown in parentheses. Categorical variables are shown as percentages and were compared by Fisher's exact test. Continuous variables are shown as mean ± SD and were compared by *t*-test or are shown as median (IQR) and compared by Mann-Whitney *U*-test. non-LVSd, not having left ventricular systolic dysfunction; LVSd, left ventricular systolic dysfunction; BMI, body mass index; AMI, acute myocardial infarction; LVEF, left ventricular ejection fraction; ACEI/ ARA, angiotensin-converting enzyme inhibitors/ angiotensin II receptor antagonists.

**Table 2:** Biochemical data of post-MI patients.

Variables	Total (25)	non-LVSd (16)	LVSd (9)	*p*-value
Hemoglobin, g/dl	13.4 ± 1.5	13.6 ± 1.5^a^	13 ± 1.4^b^	0.345
Hematocrit, %	39.4 ± 3.7	39.2 ± 3.8^c^	39.6 ± 3.9^b^	0.807
Leukocytes, mm^3^	6,495 (5,900–8,370)	6,920 (5,870–8,370)^a^	6,135 (6,010–9,240)^b^	0.301
Platelets, ×10³/mm^3^	237 ± 62	238 ± 70^a^	234 ± 51^b^	0.889
Fasting glucose, mg/dl	106 (94–115)	105 (92–113)	109 (95–132)^b^	0.610
Urea, mg/dl	37 (30–51)	34 (30–44)^a^	47 (37–53)	0.096
Creatinine, mg/dl	1.1 ± 0.3	1.0 ± 0.3	1.2 ± 0.4	0.067
Total cholesterol, mg/dl	155.6 ± 45.4	162.7 ± 49.0^b^	140.3 ± 34.8^a^	0.291
HDL-c, mg/dl	34 ± 9	35 ± 7^a^	34 ± 13^a^	0.856
LDL-c, mg/dl	83.2 ± 29.0	89.3 ± 30.8^d^	70.0 ± 21.1^d^	0.185
Non-HDL-c, mg/dl	123 ± 44	131 ± 50^a^	106 ± 24^a^	0.136
Triglycerides, mg/dl	174.5 (102.0–250.0)	115.0 (110.0-215.0)^d^	227.0 (105.0–262.0)	0.438
AST, U/l	24 (18–34)	23 (19–30)^c^	24 (18–37)^a^	0.711
ALT, U/l	29 (20–42)	30.0 (23–39)^c^	28 (19–58)^a^	0.711
Sodium, mEq/l	139 ± 5	140 ± 5^e^	137 ± 3^a^	0.334
Potassium, mEq/l	4.7 ± 0.4	4.7 ± 0.3^f^	4.7 ± 0.5^b^	0.818

The number of subjects is shown in parentheses. Continuous variables are shown as mean ± SD and were compared by *t*-test or are shown as median (IQR) and compared by Mann-Whitney *U*-test. non-LVSd, not having left ventricular systolic dysfunction; LVSd, left ventricular systolic dysfunction; HDL, high-density lipoprotein; LDL, low-density lipoprotein; ALT, alanine aminotransferase; AST, aspartate aminotransferase.

^a^ 2 missing values.

^b^ 1 missing value.

^c^ 4 missing values.

^d^ 3 missing values.

^e^ 6 missing values.

^f^ 5 missing values.

Regarding the medication, diuretic and vasodilator drugs were more frequent in patients with LVSd (*p* < 0.05), possibly because they are medications commonly used in treating this condition. The time from AMI to enrollment was longer for LVSd patients (*p* < 0.05), which highlights the fact that the development of dysfunction may be time-related. Therefore, maintenance of cardiac function monitoring after AMI may be necessary for detecting LVSd in these patients.

### miRNAs differently expressed in post-MI patients with LVSd

**Figure 1:**
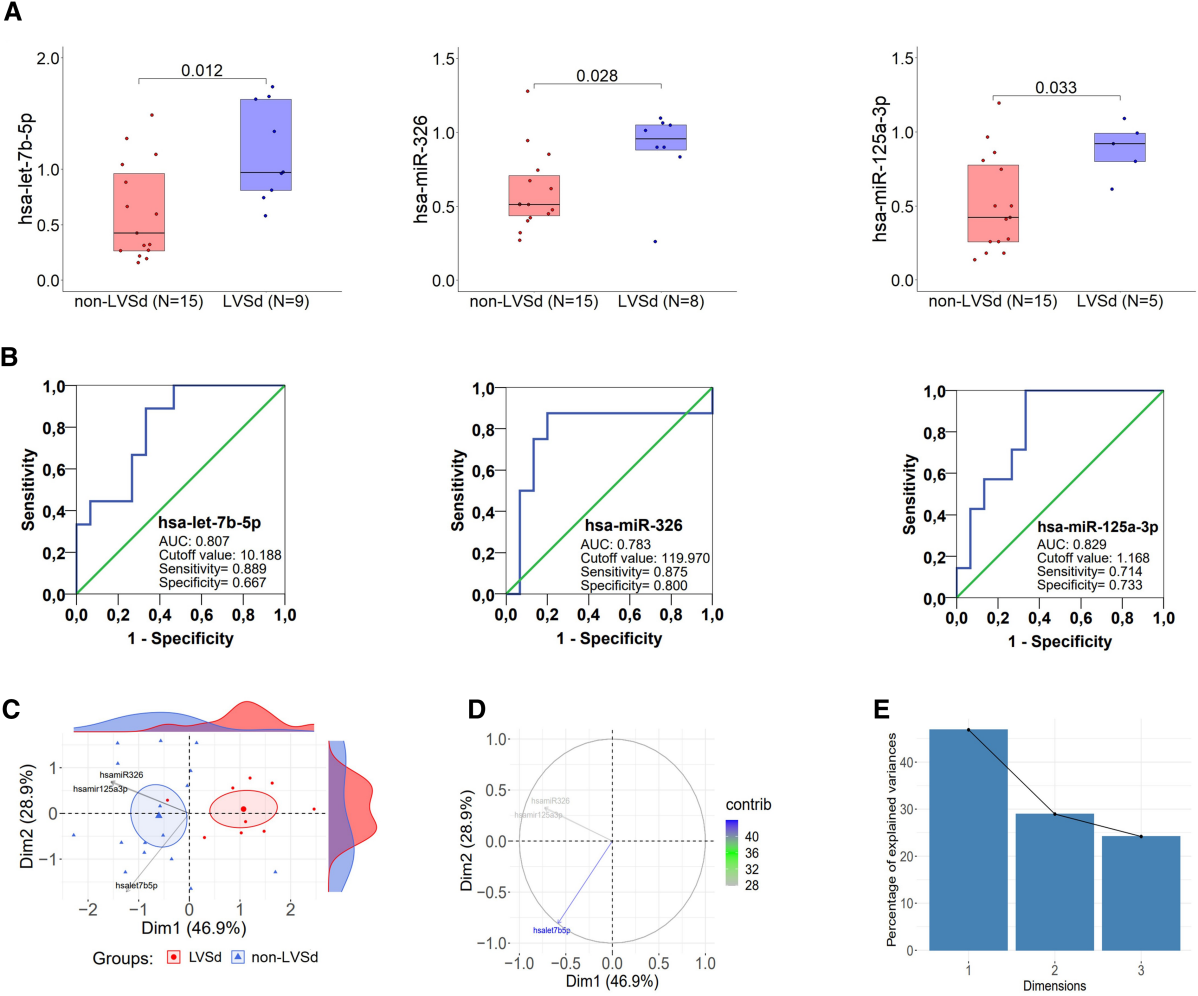
miRNAs stratify post-MI patients based on left ventricular ejection fraction. (**A**) Differentially expressed microRNAs in PBMCs between post-MI patients with left ventricular systolic dysfunction (LVSd) and non-LVSd. Data are shown as the median of relative expression (2^−^*^Δ^*^CT^×10^4^) and were compared using U-Mann Whitney; *p-value significant (<0.05). (**B**) Evaluation of the clinical value of microRNA in diagnosing left ventricular systolic dysfunction; Receiver operating characteristic (ROC) curve based on let-7b-5p, miR-326, and miR-125a-3p expression. (**C**) Principal component analysis (PCA) with spectral decomposition based on 3 different miRNAs expression shows the stratification of non-LVSd and LVSd patients. miRNAs with positive correlation point to the same side of the plot, contrasting with negatively correlated miRNAs, which point to opposite sides. Only the miRNAs highly contributing to the stratification of non-LVSd from LVSd patients are shown (let-7b-5p, miR-326, and miR-125a-3p). Small blue and red circles are concentration ellipses around the mean points of each group. The histograms aside the PCA, represent the density of the individual distribution. (**D**) Graphs of the three miRNAs obtained by PCA indicate miRNAs highly associated with post-MI patients with LVSd. (**E**) Scree plot showing eigenvalues, demonstrating the percentage of variances explained by each principal component.

The relative miRNA levels of the 61 miRNAs are shown in Supplementary Table S1. Among the miRNAs analyzed, let-7b-5p (*p* = 0.013), miR-326 (*p* = 0.028), and miR-125a-3p (*p* = 0.036) were upregulated in post-MI patients with LVSd (Figure 1A). Figure 1B shows AUC and optimal cut-off values (AUC ≥ 0.7) for three miRNAs: let-7b-5p (AUC: 0.807; 95% CI: 0.63–0.98; *p* = 0.013), miR-326 (AUC: 0.783; 95% CI: 0.54–1.00; *p* = 0.028), and miR-125a-3p (AUC: 0.800; 95% CI: 0.61–0.99; *p* = 0.036). No significant correlations among miRNAs were detected (Supplementary Table S2).

### miRNA predictors of LVSd in post-MI patients

The univariate binary logistic regression analysis with miRNA categorized according to the cut-off value showed that the high expression of let-7b-5p (OR: 16.00; 95% CI: 1.54–166.05; *p* = 0.020) and miR-326 (OR: 28.00; 95% CI: 2.42–323.70; *p* = 0.008) were able to predict LVSd in post-MI patients, but not the miR-125a-3p (OR: 10.00; 95% CI: 0.91–110.28; *p* = 0.060) (Table 3). The other variables tested were also not significant in this analysis.

**Table 3:** Univariate logistic regression analysis for predictors of LVSd in post-MI patients.

Variables	OR	95% CI	*p*-value
let-7b-5p (high expression)	16.00	1.54–166.05	**0.020**
miR-326 (high expression)	28.00	2.42–323.70	**0.008**
miR-125-3p (high expression)	10.00	0.91–110.28	0.060
Sex (male)	0.81	0.11–6.04	0.835
Diabetes	6.00	1.00–35.91	0.050
Smoking	0.75	0.14–3.94	0.734
Alcohol intake	0.42	0.07–2.36	0.323
Body mass index	0.98	0.83–1.17	0.845
Time from AMI to enrollment	1.00	1.00–1.00	0.102

Bold indicates a significant *p*-value (< 0.05).

High expression was considered the miRNA optimal cut-off value. LVSd, left ventricular systolic dysfunction; OR, odds ratio; CI, confidence interval; AMI, acute myocardial infarction.

The multivariate models were built up with the let-7b-5p and miR-326 individually, and adjusted by sex, body mass index, smoking, alcohol intake, diabetes, and time from AMI to enrollment (Table 4). For both models, only let-7b-5p and miR-326 remained significant by the forward stepwise method, demonstrating their robust predictive capacity independently of the characteristics of the participants.

**Table 4:** Multivariate logistic regression analysis for predictors of LVSd in post-MI patients.

Variables	OR	95% CI	*p*-value
**Model 1**			
let-7b-5p (high expression)	16.00	1.54–166.05	**0.020**
**Model 2**			
miR-326 (high expression)	28.00	2.42–323.70	**0.008**

Bold indicates a significant *p*-value (< 0.05).

Multivariate logistic regression analysis was performed using a forward stepwise method only with statistically significant miRNAs in the univariate analysis and adjusted by sex, body mass index, cigarette smoking, alcohol intake, diabetes, and time from acute myocardial infarction to enrollment as independent variables. High expression was considered the miRNA optimal cut-off value. LVSd, left ventricular systolic dysfunction; OR, odds ratio; CI, confidence interval.

### miRNAs expression stratifies post-MI patients according to LVSd

Next, we performed principal component analysis (PCA) to examine the association between the expression of miRNAs and individuals (observations) while stratifying groups based on the level of the miRNA. This analysis indicated that miRNAs expression levels could be used to stratify post-MI patients according to LVSd (Figures 1C–E). The miRNAs miR-326, miR-125a-3p, and let-7b-5p seemed to play a significant role in stratifying post-MI patients by LVSd. This analysis indicated that these three miRNAs could be biomarkers for post-MI outcomes.

### miRNAs-targets are involved with immune response and cardiovascular pathogenesis in post-MI patients with LVSd

**Figure 2:**
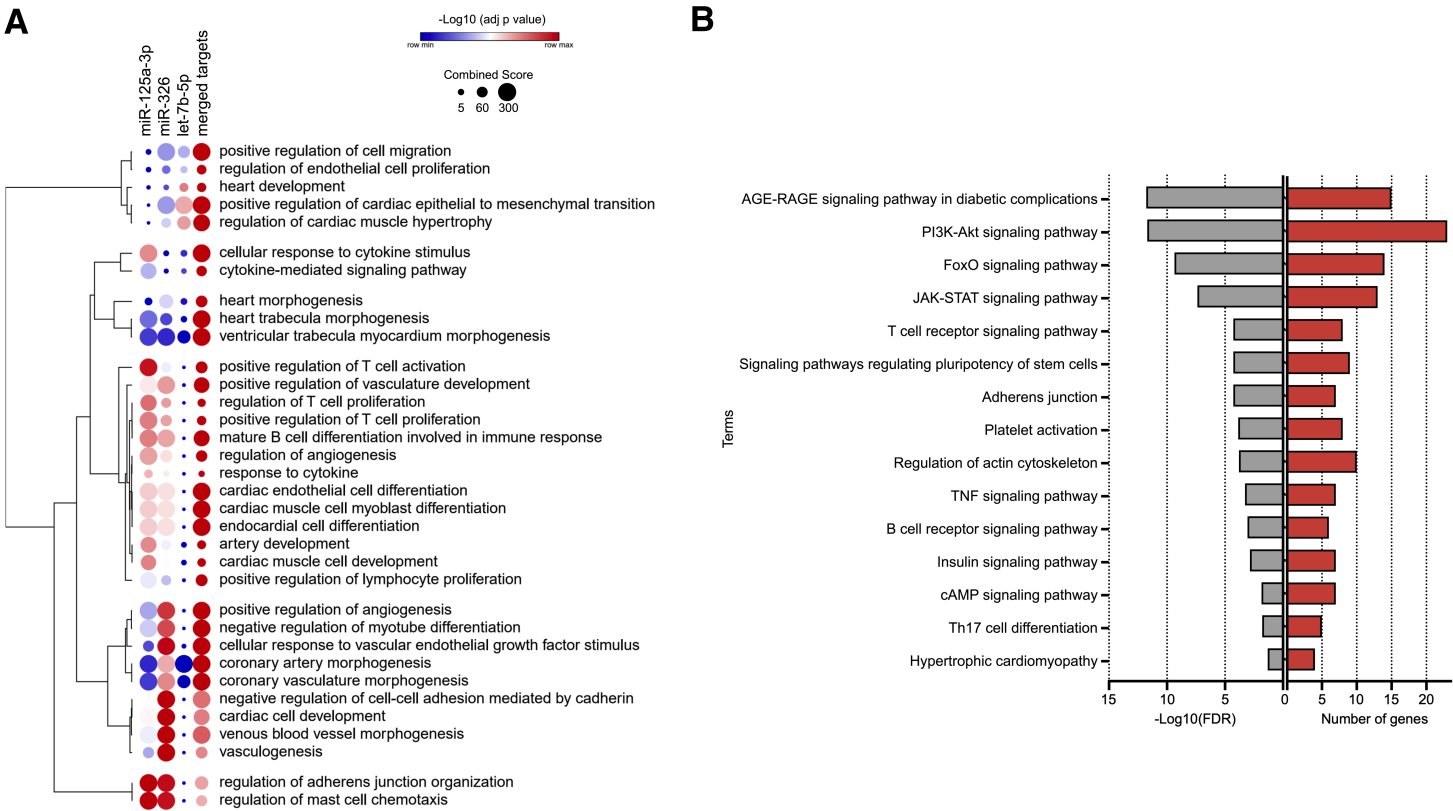
The gene ontology (GO) and Kyoto encyclopedia of genes and genomes (KEGG) pathway enrichment analyses for the validated targets of microRNAs predictors of left ventricular dysfunction in post-myocardial infarction patients; (**A**) terms of GO biological processes related to adverse cardiac remodeling; (**B**) terms of KEGG pathways related to adverse cardiac remodeling.

Based on strong validated evidence of the two miRNA-target tools, we found 91 target genes of let-7b-5p, miR-326, and miR-125a-3p (Supplementary Table S3). Results were obtained by enrichment analysis based on the Gene ontology (GO) and Kyoto Encyclopedia of Genes and Genomes (KEGG) databases (Supplementary Tables S4, S5). Among the GO biological processes (Figure 2A), terms related to adverse cardiac remodeling were evidenced, such as positive regulation of cardiac epithelial to mesenchymal transition, regulation of cardiac hypertrophy, regulation of adherent's junction organization, cardiac endothelial cell differentiation, regulation of angiogenesis, cytokine-mediated signaling pathway, positive regulation of T cell proliferation, positive regulation of T cell activation, regulation of mast cell chemotaxis.

The KEGG pathway enrichment analysis (Figure 2B) revealed enriched signaling pathways, such as those that regulate the expression of growth factors, cytokines, cell proliferation, and apoptosis, including the AGE-RAGE signaling pathway in diabetic complications, PI3K-Akt signaling pathway, FoxO signaling pathway, and JAK-STAT signaling pathway. In addition, pathways related to immune response, cardiac hypertrophy, and adherents' junctions also appeared statistically enriched.

**Figure 3:**
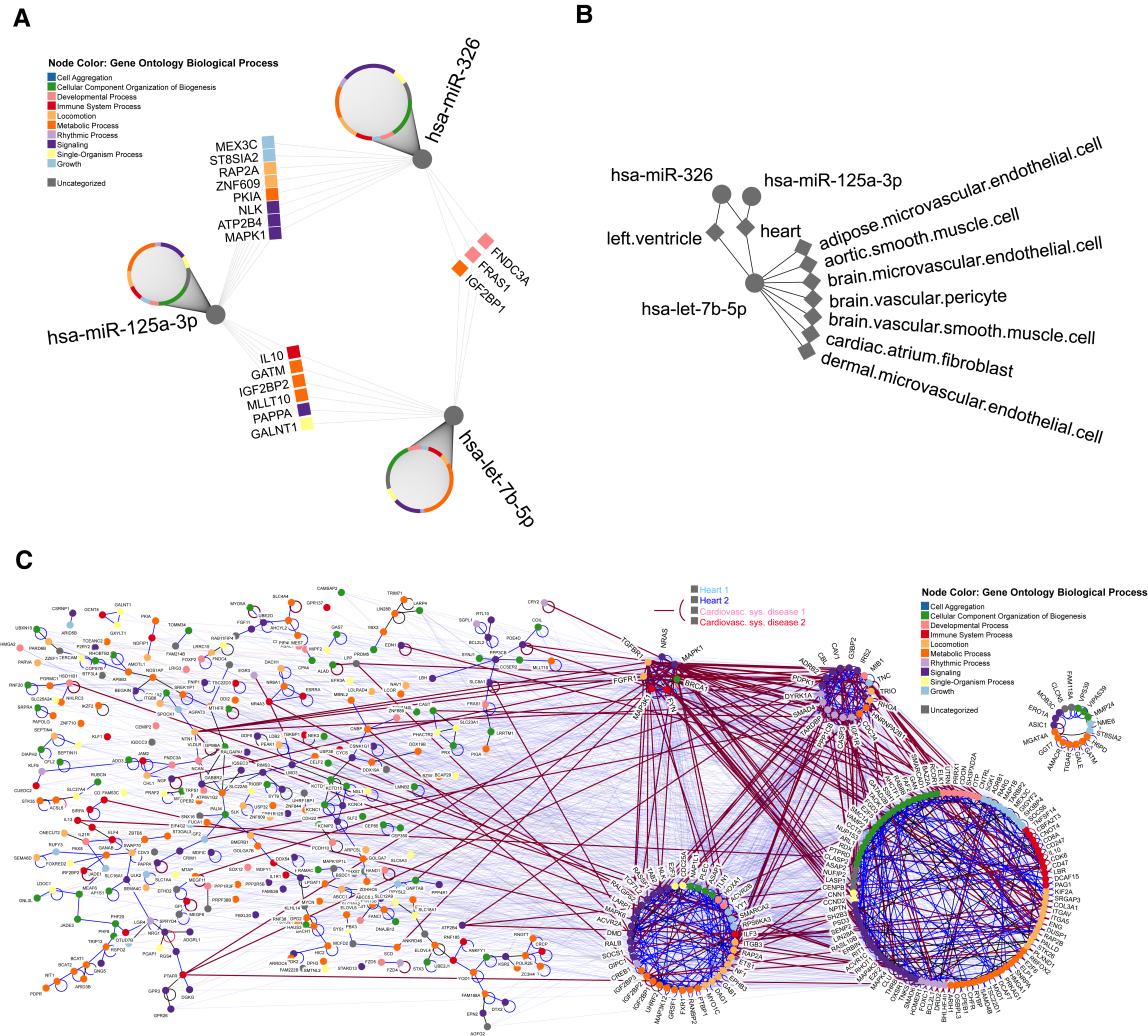
Molecular network of miRNA-mRNA interactions. The miRNA-gene network was prepared using the top 1% of gene targets. (**A**) Gene Ontology (GO) biological process across all gene targets, as per color legend. Only shared targets are highlighted with names. (**B**) Tissues associated with the three miRNAs. (**C**) Physical protein interaction network among miRNA targets. All direct human interactions among the target genes are shown, highlighting those relevant to heart and cardiovascular disease. With edge thickness, transparency, and color we differentiate whether only one of the interactors has the annotation (color as per legend and partially transparent) or both interacting partners share the annotation (color as per legend and strong edge) and whether the annotation is valid for both heart and cardiovascular system disease. In addition, node color corresponds to the GO biological process.

Finally, to better understand the involvement of these three miRNAs with cardiovascular pathogenesis, we performed an interaction analysis with the target genes related to cardiac processes. This analysis revealed an interconnected network of target genes modulated by let-7b-5p, miR-326, and miR-125a-3p (Figures 3A–C). Figure 3A displays the overlapping target genes of the three miRNAs, indicating a complex combination of target multiplicity and miRNA cooperativeness on the LVSd. Meanwhile, Figure 3B shows the relation between miR-326 and the left ventricle and the association of miR-125a-3p and let-7b-5p with the heart and left ventricle. Figure 3C displays the interactions of these miRNA targets represented by their physical protein-protein interaction (PPI) and gene ontology (GO) relationships.

## Discussion

The present study investigated the expression of miRNAs from PBMCs and found that let-7b-5p, miR-326, and miR-125a-3p were upregulated in LVSd patients, highlighting let-7b-5p and miR-326 as independent predictors of cardiac dysfunction in post-MI patients. Thus, this work expands the molecular mechanisms involved in post-MI cardiac remodeling, since the role of miRNAs in this condition remains poorly explored. Particularly, LVSd is a consequence of adverse cardiac remodeling, which affects approximately 40% of patients with STEMI and is associated with increased morbidity and mortality. Myocardial infarction impacts the immune system and causes changes in the blood transcriptome landscape, which relate to the development of LVSd in post-MI (24, 25). Thus, a better understanding of the involvement of essential modulators of gene expression, such as let-7b-5p, miR-326, and miR-125a-3p is fundamental to identifying new therapeutic targets and disease biomarkers.

Several transcriptomic changes have been investigated in post-MI patients, showing the immune system's role during cardiac remodeling (25, 26). In this context, our results suggest a dysregulated expression pattern of the miRNAs let-7b-5p, miR-326, and miR-125a-3p, with involvement in immunological pathways (i.e., regulation of mast cell chemotaxis, positive regulation of T cell proliferation), cell-cell communication (i.e., regulation of adherens junction organization, cytokine-mediated signaling pathway), and cardiac changes (i. e., regulation of cardiac hypertrophy, regulation of angiogenesis). The let-7b-5p is a well-studied miRNA that plays an essential role in targeting important genes or pathways involved in the development of cardiovascular diseases (27–29). This miRNA was found in pericardial fluid exosomes and was able to restore the angiogenic ability of endothelial cells, as well as improve post-ischemic blood flow and angiogenesis in mice (30). Interestingly, Kuosmanen et al. (31) reported that let-7b-5p is one of the five most abundant miRNAs in the pericardial fluid of HF patients and Marques et al. (32) described the expression of cardio-miRNAs in the transcardiac gradient of advanced HF patients, showing that the failing heart releases let-7b. These findings are in agreement with our study, suggesting that cardiac injury alters the expression of let-7b-5p and is related to HF's pathophysiology.

Another dysregulated miRNA identified was miR-326, which has an essential modulatory function in the immune system (33), and a relationship with various autoimmune diseases (34, 35). In the cardiovascular context, Danaii et al. (36) showed that the levels of miR-326 from PBMC were increased in slow coronary flow patients, and Dolati et al. (37) report its expression was upregulated in elderly patients with ischemic stroke associated with disease severity and IL-17 production. To the best of our knowledge, our study is the first to demonstrate the differential expression of miR-326 in PBMCs of post-MI patients. In addition to the importance of miR-326 in immune regulation, this study showed that this miRNA appears to regulate the angiogenesis process, targeting essential genes, such as FGF1 and VEGF. Similar findings were reported by Li et al. (38), who described that miR-326 improved the cardiac function of ischemic hearts through enhanced angiogenesis.

It has been shown that miR-125a-3p reduced vascular smooth muscle cell growth and migration, inhibiting the occurrence of vascular stenosis (39) and that miR-125a-3p promotes cellular apoptosis in a model of atherosclerosis pathogeny treated with ox-LDL (40). The expression of miR-125a-3p was investigated in failing heart, but no statistical difference was found when compared with healthy controls (41). However, intravenous injection of miR-125a-3p adenovirus improved cardiac function and fibrosis while reducing inflammatory responses in mice with diabetic cardiomyopathy (42). The high expression of miR-125a-3p in post-MI patients seems to be involved in the cellular response to cytokine stimulus, regulation of mast cell chemotaxis, and other biological processes involving activation, proliferation, and differentiation of immune cells, which can explain the cardiac remodeling and worse prognosis in these patients.

Noteworthy, these miRNAs may regulate signaling pathways involved in cardiac dysfunction. The AGE-RAGE signaling pathway is a key mechanism responsible for increased matrix contraction and myofibroblast differentiation, with stiffening of the left ventricle and poor heart function in diabetics (43). The PI3K-Akt signaling pathway activation has been shown to promote cardioprotection after AMI, and reduced PI3K-Akt expression led to myocardial fibrosis and reduced left ventricular function (44). The pivotal role of the FoxO signaling pathway in the autophagic activity induced by the pathological process has been related to the development of HF (45). In contrast, the protective role of FoxO transcription factors was shown in response to acute ischemia/reperfusion injury by limiting ROS production and cell death in the heart (46). JAK/STAT pathway is another important pathway enriched in our analysis, which has been related to the apoptotic response after MI and implicated in promoting myocardial angiogenesis, improving cardiac function (47).

Despite the interesting findings, this study has some limitations. The sample size was limited, requiring a larger sample to validate the findings of this study. Other limitation is that the ventricular dysfunction was analyzed in post-MI patients regardless of HF symptoms. Therefore, it is important to explore the role of let-7b-5p, miR-326, and miR-125a-3p in HF patients and disease progression. Also, as only PBMC miRNAs were analyzed, inferences about their direct role in the cardiac structure are limited. Further studies with analysis of miRNAs in cardiac tissue may provide a more comprehensive understanding of their role in cardiac remodeling.

In conclusion, this study demonstrates that let-7b-5p, miR-326, and miR-125a-3p are upregulated in PBMCs from patients with post-MI LVSd. These miRNAs appear to regulate pathways involved in cardiovascular pathogenesis, suggesting the involvement of the immune system in the pathophysiology of cardiac dysfunction through the differentiated expression of miRNAs. Furthermore, this study demonstrates that the expression of let-7b and miR-326 can predict LVSd and support future research that validates their potential application as LVSd biomarkers in post-MI patients.

## Data Availability

The original contributions presented in the study are included in the article/**Supplementary Material**, further inquiries can be directed to the corresponding author.
