# Supplementary material for: The let-7b-5p, miR-326, and miR-125a-3p are associated with left ventricular systolic dysfunction in post-myocardial infarction
Source: Front Cardiovasc Med. 2023 May 12;10:1151855. doi: 10.3389/fcvm.2023.1151855 (PMC10218134; doi:10.3389/fcvm.2023.1151855)
Supplement: Supplementary file 1 [file Image1.pdf]

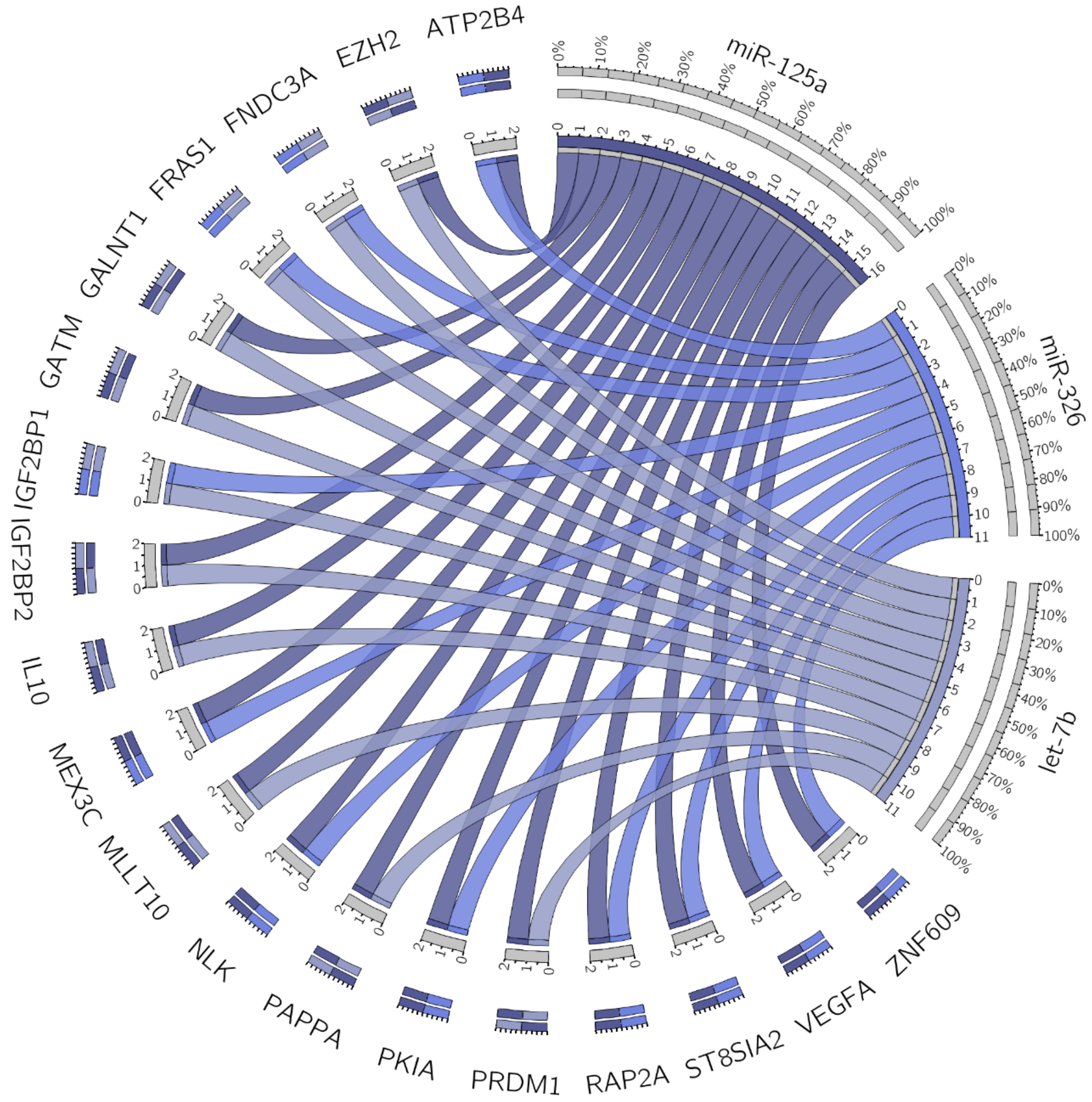

Supplementary Figure S1: Network visualization of shared targets of the three miRNA (let-7b, miR-125a, and miR-326), using Circos software.
